# Carotid intima-media thickness is a relatively inexpensive and favorable prognostic marker in patients with spondyloarthritis 

**DOI:** 10.1590/1516-3180.2013.1316728

**Published:** 2013-12-01

**Authors:** Sevket Balta, Sait Demirkol, Ugur Kucuk, Zekeriya Arslan, Murat Unlu, Vusal Veliyev

**Affiliations:** I MD. Attending Physician, Department of Cardiology, Gulhane Medical Faculty, Ankara, Turkey.; II MD. Adjunct Professor, Department of Cardiology, Gulhane Medical Faculty, Ankara, Turkey.; III MD. Doctoral Student, Department of Cardiology, Gulhane Medical Faculty, Ankara, Turkey.

Dear Editor,

We read the article "Carotid intima-media thickness (cIMT) in spondyloarthritis patients" by Skare et al.^1^ They aimed to investigate cIMT in spondyloarthritis patients and correlate this with clinical parameters and inflammatory markers. They concluded that spondyloarthritis patients have a higher degree of subclinical atherosclerosis than controls, thus supporting clinical evidence of increased cardiovascular risk in rheumatic patients. We believe that these findings will be guides for further studies about subclinical atherosclerosis on cIMT inpatients with spondyloarthritis. 

Carotid intima-media thickness is a widely accepted marker for subclinical atherosclerosis.^2^ Increased cIMT is a common indicator of atherosclerotic involvement of the vascular structure, thereby indicating coronary artery disease, cerebrovascular disease and peripheral arterial disease. cIMT has received increased attention due to its role as an independent prognostic factor for hypertension, chronic kidney disease, diabetes and systemic inflammatory diseases such as systemic lupus erythematosus, Behçet and psoriasis.^3^ It is a non-invasive method for assessing endothelial dysfunction in clinical practice. Previous reports have suggested that cIMT is a useful sonographic marker for early atherosclerosis. Hence, cIMT assessment can be used to document regression or progression of atherosclerosis, and it can be used to make correlations with other inflammatory markers. 

Chronic obstructive pulmonary disease (COPD) and atherosclerosis may occur due to similar risk factors, and may be significant causes of morbidity and mortality. Obstructive sleep apnea is associated with elevated risk of cardiovascular events. The risks of early atherosclerosis and cardiovascular events in adults with COPD are higher, independent of risk factors. cIMT shows direct proportionality with age and inverse proportionality with FEV1%, and is a non-invasive, easily applicable and cheap method that can be used in determining the risk of atherosclerosis.^4^

The significant acceleration of atherosclerotic change in hypothyroid patients may be explicable by several mechanisms. Thyroid hormone changes occur in the presence of a variety of major risk factors for atherosclerosis, such as altered serum lipid profiles, high blood pressure, obesity and hypercoagulable state. Hypothyroidism has a more adverse cardiovascular risk profile and greater cIMT. Nonalcoholic fatty liver disease (NAFLD) is characterized by excessive accumulation of fat in the liver cells. It is strongly associated with cardiovascular risk factors for atherosclerosis. Obese adolescents with NAFLD and subclinical hypothyroidism have been found to present a more adverse cardiovascular risk profile and greater cIMT.^5^

In conclusion, although cIMT is a novel, simple, inexpensive and noninvasive method for evaluating endothelial dysfunction and subclinical atherosclerosis in clinical practice, it may be affected by many conditions. Therefore, without other inflammatory markers, cIMT alone may not provide information to clinicians about the prognosis in patients with spondyloarthritis.

## RESPONSE TO LETTER TO THE EDITOR

**Thelma Larocca Skare ^I^** **; Guilherme Cortez Verceze ^II^** **; André Augusto de Oliveira ^II^** **; Sonia Perreto ^III^**

^I^MD, PhD. Head of Rheumatology Unit, Hospital Universitário Evangélico de Curitiba, Paraná, Brazil ^II^Student, School of Medicine, Faculdade Evangélica do Paraná (Fepar), Curitiba, Paraná, Brazil ^III^MD. Cardiologist, Echocardiography Service, Hospital Universitário Evangélico de Curitiba, Curitiba, Paraná, Brazil

We thank the authors of the letter *"Carotid media thickness is a relatively inexpensive and favorable prognostic marker in patients with spondyloarthritis"* for their interest in our paper.^1^

We agree with and reinforce the observation that carotid intima-media thickness (cIMT) measurement is a very well-accepted marker of subclinical atherosclerosis in a variety of conditions,^2^ from rheumatic diseases to chronic pulmonary obstructive diseases and hypothyroidism. We also agree that it has the advantage of being inexpensive and non-invasive, which makes it possible to use it repeatedly to monitor a patient's condition.

As pointed out in the letter, a large variety of clinical situations can affect the results from cIMT measurements. Accordingly, we were careful to exclude patients with other inflammatory conditions in our work, even at the expense of reducing the size of the sample. Also, at our service, thyroid function tests are done in all patients with inflammatory diseases as part of a routine protocol. Besides this, we included a control group that was as close as possible to the group of patients with spondyloarthritis. This practice allowed us to become confident in ascribing the observed changes in cIMT measurements to the pathophysiological process of this rheumatic disease.

We share with our colleagues the frustration of not being able to correlate the changes seen in cIMT with finding of high inflammatory markers. However, some peculiarities in measuring inflammatory process in this group of rheumatic diseases should be taken into account.

Firstly, acute-phase reactant tests (such as C-reactive protein and erythrocyte sedimentation rate) have limited value for measuring spondyloarthritis inflammatory activity^3^ and no surrogate markers have yet been found. In our study, we also used composite measurements such as BASDAI (Bath Disease Activity Index),^4^ which, although much used in daily practice and well accepted by the rheumatology community, has its own problems: it is a test that relies only on information provided by the patient, gives high value to pain symptoms and thus may be influenced by the momentary psychological status. 

Secondly, we need to emphasize that spondyloarthritis is a highly chronic disease (the mean length of time with the disease among our patients was nearly ten years). The findings from measurements on cIMT may reflect the sum of past inflammatory events suffered by the patient that are not currently seen.

Our results showing increased cIMT measurements in spondyloarthritis patients are in line with those of Mathieu et al.,^5^ who showed that spondyloarthritis patients are at higher risk of many types of cardiovascular diseases. Awareness of these complications is important for good care of the patients.
